# Dynamic and static properties of stadium-shaped antidot arrays

**DOI:** 10.1038/s41598-020-77074-2

**Published:** 2020-11-18

**Authors:** E. Saavedra, R. M. Corona, N. Vidal-Silva, J. L. Palma, D. Altbir, J. Escrig

**Affiliations:** 1 Departamento de Física, Universidad de Santiago de Chile, Avda. Ecuador 3493, 9170124 Santiago, Chile; 2 Departamento de Ciencias Físicas, Universidad de La Frontera, Casilla 54-D, 4811186 Temuco, Chile; 3 Universidad Central de Chile, Avda. Santa Isabel 1186, 8330601 Santiago, Chile; 4 Center for the Development of Nanoscience and Nanotechnology, Avda. Libertador Bernardo O’Higgins 3363, 9170124 Santiago, Chile

**Keywords:** Materials science, Nanoscience and technology, Physics

## Abstract

In this work we performed a detailed numerical analysis on the static and dynamic properties of magnetic antidot arrays as a function of their geometry. In particular, we explored how by varying the shape of these antidot arrays from circular holes to stadium-shaped holes, we can effectively control the magnetic properties of the array. Using micromagnetic simulations we evidenced that coercivity is very sensitive to the shape of antidots, while the remanence is more robust to these changes. Furthermore, we studied the dynamic susceptibility of these systems, finding that it is possible to control both the position and the number of resonance peaks simply by changing the geometry of the holes. Thus, this work provides useful insights on the behavior of antidot arrays for different geometries, opening routes for the design and improvement of two-dimensional technologies.

## Introduction

Low-dimensional magnetism is an emergent field in condensed matter physics whose study and understanding have provided many novel phenomena that allow the improvement of potential spintronics, magnonics, electronic, and microwave devices. In this context, magnetic thin films with periodic arrays of holes, the so-called antidot arrays, have concentrated current attention since they allow exploring a broad range of applications, such as a new generation of transistors^1^, sensors^2,3^, and ultra-high density recording media^4^. This attention is due to the absence of the superparamagnetic limit since there are no isolated magnetic islands^5^. More recently, magnonic cryslals^6^ made from antidot arrays^7–10^, which can be considered as meta-materials that allow or forbid the propagation of spin waves, have gained a lot of attention in the field of topological magnonics, where antidots are used to control the spin wave bandgap in the system, as well as the topological nature that could emerge. In fact, the static and dynamic properties of magnetic antidot arrays are currently being investigated for use in potential applications, such as microwave based devices^11,12^ and filters^13^, applications on microfluidic devices^14^, anisotropic magnetoresistance based sensors^15^, and in transistors^1^ and diodes^16^ for low power electronics applications.

From the experimental point of view there are several techniques for obtaining magnetic antidot arrays such as e-beam^2,17^, UV^18^ and colloidal^19^ lithography, porous anodic alumina^20,21^, block copolymer templates^22^, nanochannel glass^23^ and focused ion beam (FIB) patterning^24–26^, among others. Regarding the features that antidots possess, it has been widely reported that static properties such as remanence^27^, coercivity^27,28^, and the easy-axis magnetic anisotropy^28–31^, can be controlled by modifying the hole size, the distance between them, and the material used to fabricate the array. Therefore, the appearance of different domain structures, magnetoresistance effects, distinct magnetization reversal processes and modifications on the equilibrium magnetic configurations when changing the shape, size, and density of antidot holes have been deeply studied^17,32–36^. The way in which the holes are arranged also has a direct incidence on the static properties of the array^36–38^.

On the other hand, the dynamic properties of magnetic antidot arrays are also an important issue when having in mind these systems for future applications. It is crucial to notice that the term *dynamic* encodes two temporal scales according to the amplitude of spin motion: while large amplitudes are related to magnetization reversal processes by means of a constant magnetic field, small amplitudes are typically encountered in ferromagnetic resonance (FMR) experiments, which ultimately refer to the excitation and propagation of spin waves^39–41^. This technique probes the magnetization dynamics of the samples using microwave fields whose maximum absorption occurs when the microwave frequency matches the frequency of the resonance modes of the system^42^. A central quantity of the ferromagnetic resonance is the dynamic magnetic susceptibility, which can be obtained by micromagnetic simulations^43–52^. Numerical studies on the dynamic susceptibility of antidot arrays have shown that by modifying the hole density or hole geometry, it is possible to achieve a reasonable degree of control over the number of resonant peaks, their amplitude and their position^53,54^. In fact, it has been reported that antidot arrays with holes of different shape and size substantially modify the spin wave spectra of these systems^55–57^, establishing the geometrical variation of the antidots as the key for the tunability of spin wave structures. Furthermore, not only the shape and density of the antidot arrays play an important role in the dynamic properties of these structures, but also the lattice ordering and its symmetry have proven to be a feasible way to control and modulate the spin wave spectra^57–59^. Thus, if we change the shape of the holes in an antidot array, both the static and dynamic properties of the system will be affected. The physics behind this can be understood in terms of changes on the demagnetizing field when we vary the shape of the holes^60^. Since this field, usually known as the shape anisotropy field, depends entirely on the shape of the sample, it is expected to change, also affecting the effective field of the system.

As stated above, there are a number of previous works in which the dependence of the spin wave spectra and the static properties of antidot arrays as a function of the size and shape of their holes has been studied. In most of these, the static studies were restricted to the coercivity behavior when changes in the magnetostatic field were introduced modifying the size and shape of the holes. However, a systematic study on how the coercivity, remanence, and magnetization reversal modes vary when the holes size is varied along one direction is still necessary to understand the role that the shape anisotropy plays on such structures, with the ultimate goal of achieving better control of the static properties of these arrays.

On the other hand, regarding the dynamic studies, most of them were performed by exciting the spin wave modes on a saturated state in the presence of a bias field. This is an important issue because the excitation of spin wave modes in a homogeneous magnetic background is substantially distinct to the textured background case. In the first case, an excitation with a small magnetic pulse perpendicular to the bias magnetic field gives rise to distinct resonant modes. However, when the pulse is applied along the same direction as the bias field, no spin wave mode is excited, according to linear spin wave theory^61^. Furthermore, in realistic systems, although a significant large bias field saturates the sample, there will always be small regions with non-uniform magnetization that allows the activation of the dynamics but in such cases with much less efficiency. On the other hand, the presence of magnetic textures, such as those that appear for the minimum energy configuration of an antidot array at zero bias field, allows the excitation of different spin waves modes depending on the direction in which the magnetic pulse is applied. These modes emerge from the non-collinearity of the magnetic moments and also depend directly on the effective field of the system. This case is characterized by the existence of resonance series due to spatially non-uniform spin wave modes^62^. Thus, the study and control of these modes, in the presence of a textured background at zero bias field, is a topic that deserves special attention. Therefore, we should notice that any change in the effective field will give rise to modifications in the static and dynamic properties of the system.

**Figure 1:**
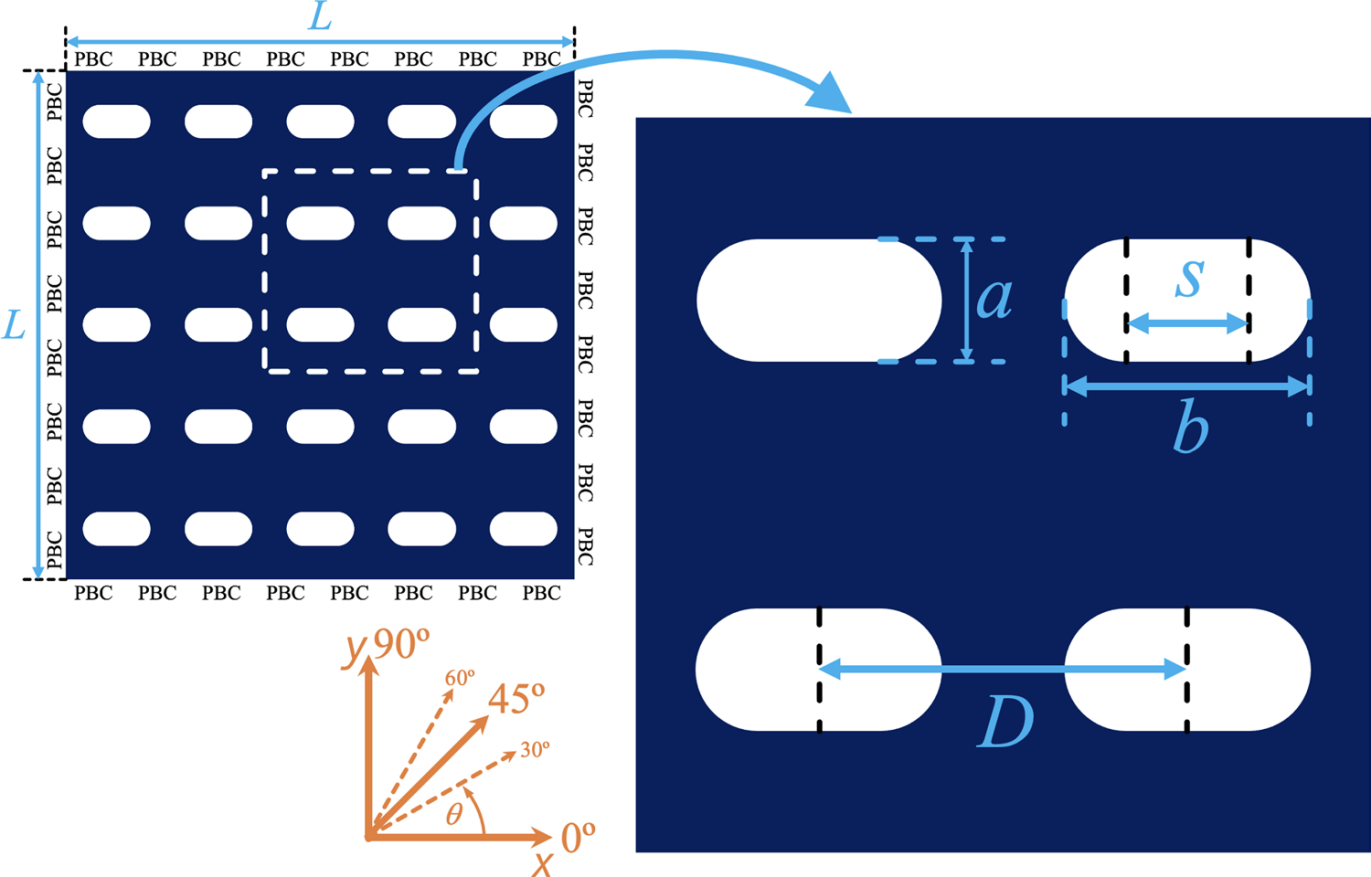
(Color online) Schematic representation of a stadium-shaped antidot array. The constant magnetic field ${\mathbf {H}}$ is applied along the angle $\theta$.

Based on previous ideas, in this paper we study how both the static and dynamic properties of antidot arrays are affected when the effective field of the sample changes. These changes are introduced by systematically modifying the geometry of the holes, from circular to stadium-shaped antidot arrays (see Fig. 1), allowing us to modulate the demagnetizing field of the sample. Our aim is to better understand the physical origin of the observed changes in static and dynamic properties when a shape anisotropy is gradually induced. In general, despite the idea of modifying the demagnetizing field by changing the shape of holes is not new^63^ we exploit the mechanism of elongating the holes to induce an easy-axis anisotropy in the absence of bias magnetic field to effectively study how the shape of holes affects the mentioned properties of the system.

## Micromagnetic simulations

In this section we set the magnetic and geometric parameters used in our micromagnetic simulations. As depicted in Fig. 1, we consider a magnetite $(\text {Fe}_3\text {O}_4)$ thin film, with periodic boundary conditions, containing an antidot array. The size of our squared system measures $L = 1.5$ $\mu$m along the *x* and *y* directions, while the film thickness is $w = 10$ nm. On the other hand, the minor axis of the holes measures $a = 100$ nm and the major axis *b* varies between 100 and 290 nm, the inter-hole spacing is $D = 300$ nm, so then for $b = a$ we have circular antidots, while for $b>a$ we progressively move towards stadium-shaped antidots, which are arrays of holes made up of a rectangle with sides *a* and *s*, and two half-circles with diameter *a* (see Fig. 1). The sizes considered in our simulations are similar to some experimentally studied arrays^25,26,41,60,64,65^.

We use the Object Oriented MicroMagnetic Framework (OOMMF) public code^66^, with periodic boundary conditions (PBCs)^67,68^, to numerically solve the Landau–Lifshitz–Gilbert (LLG) equation and explore both the static and dynamic properties mentioned above. The magnetic parameters used in the simulations^69^ are a saturation magnetization $M_s=480\times 10^3\ {\mathrm{A}} \ {\mathrm{m}}^{-1}$ and a exchange constant $A=15.3\times 10^{-12}\ {\mathrm{J}} \ {\mathrm{m}}^{-1}$. Theses parameters represent magnetite, $\hbox {Fe}_3$ $\hbox {O}_4$, a soft magnetic material that allows us to observe in a better way the contribution of the geometric parameters. Therefore we neglected the magnetocrystalline anisotropy. The energy considered in the system is given by

1 $$\begin{aligned} E_{m}[{\mathbf {M}}] = E_{\text {ex}} + E_{\text {dem}} + E_{\text {z}}\,, \end{aligned}$$

where $E_{\text {ex}}$, $E_{\text {dem}}$, $E_{\text {z}}$ correspond to the exchange, dipolar and Zeeman energies, respectively, which implicitly define the effective field as $\mu _0{\mathbf {H}}_{\text {eff}} = -\delta E_{m}[{\mathbf {M}}]/\delta {\mathbf {M}}$, being $\delta /\delta {\mathbf {M}}$ the variational derivative respect to the magnetization ${\mathbf {M}}$ and $\mu _0$ the vacuum susceptibility. Finally, and in order to obtain results in a reasonable time, we have discretized the system into cubic cells of $5\times 5\times 5$ nm$^3$, that is smaller than the magnetite exchange length $l_{ex}^{\text {Fe}_3\text {O}_4}= \left( 2A/\mu _0M_s^2\right) ^{1/2} = 10.28$ nm^70^, satisfying the premise of micromagnetism, which states that the cell size must be smaller than the exchange length.

To study the static properties of antidot arrays, we used a damping parameter $\alpha =0.5$ for all simulations and an external magnetic field *H*, applied in the $x-y$ plane, that forms a $\theta$ angle with the *x*-axis. The external magnetic field is applied until a saturated state is achieved for $H\approx 1.5$ kOe. Next, the magnetic field is reverted, obtaining thus the hysteresis loop. This is done for selected values of *b* and for different values of the angle $\theta$, which allows us to obtain a global picture of how the demagnetizing field, associated with the geometry of the holes, affects the magnetic properties of the antidot array.

On the other hand, for the study of the dynamic properties, we used $\alpha = 0.025$, a value previously used in the literature^53,71^ and which is small enough to avoid an overdamped system, but that allows us to capture the essence of dynamics (see also Fig. S1 of Section I in the Supplementary Information for further details). Furthermore, we used the Ringdown method^42^ to simulate the FMR spectrum, a method that considers the following steps: first, the minimum energy configuration of the system is obtained, by minimizing the total energy $E_{m}$ given by Eq. (1) in the absence of an external magnetic field ($E_{\text {z}}=0$), which gives rise to the states displayed in Fig. 5. Then, this equilibrium configuration is excited with a small magnetic field of the form $h(t)=1000 \exp (-10^9 t)$ A/m^51,52^, which allows obtaining the temporal evolution of the magnetization of the system by numerically solving the LLG equation considering all the energy terms, $E_{m}$. The amplitude of this pulse must be small enough to keep the system in the linear response regime^72^. The temporal evolution of the magnetization under the action of the exciting field is collected for 30 ns recording the magnetization configuration at uniform time intervals of 10 ps allowing a spectral resolution of 0.033 GHz. Then, the small exciting magnetic field *h*(*t*) and the magnetization distribution *M*(*r*, *t*) are transformed to the frequency domain $\left[ h(\omega ),M(\omega )\right]$ using the fast Fourier transform (FFT) method. The dynamic susceptibility, which corresponds to the imaginary part of the magnetic susceptibility, is calculated by dividing the Fourier transform of the response $M(\omega )$ by the Fourier transform of the excitation $h(\omega )$^73^. Finally, in order to confirm the origin of the resonant peaks, we can reconstruct the spatial profiles of the resonant modes by calculating the temporal Fourier image for each site as^74^ ${\tilde{m}}\left( r_{ijk},f_n\right) = \text {DFT}_t\left( m\left( r_{ijk},t\right) \right)$, where $\text {DFT}_t$ is the discrete-time Fourier transform, the subscript *ijk* corresponds to the spatial coordinates *x*, *y*, *z* of each cell, and the subscript *n* indicates the position of the frequency in the power spectra. These images are essentially the profiles of the magnetization for any particular frequency.

## Results

In this section we show and analyze the results of our micromagnetic simulations for both the static and dynamic properties of stadium-shaped magnetic antidot arrays. For the static behavior we focus on the angular dependence of the coercivity, remanence, and magnetization reversal modes for different values of *b*, which controls the geometry of the holes. On the other hand, for the dynamics we focus on the dynamic susceptibility and the frequency of resonance peaks as a function of *b*. We also study the spatial distribution of the dynamic susceptibility both when the direction of the applied magnetic field varies and when *b* varies.

### Static magnetic properties

**Figure 2:**
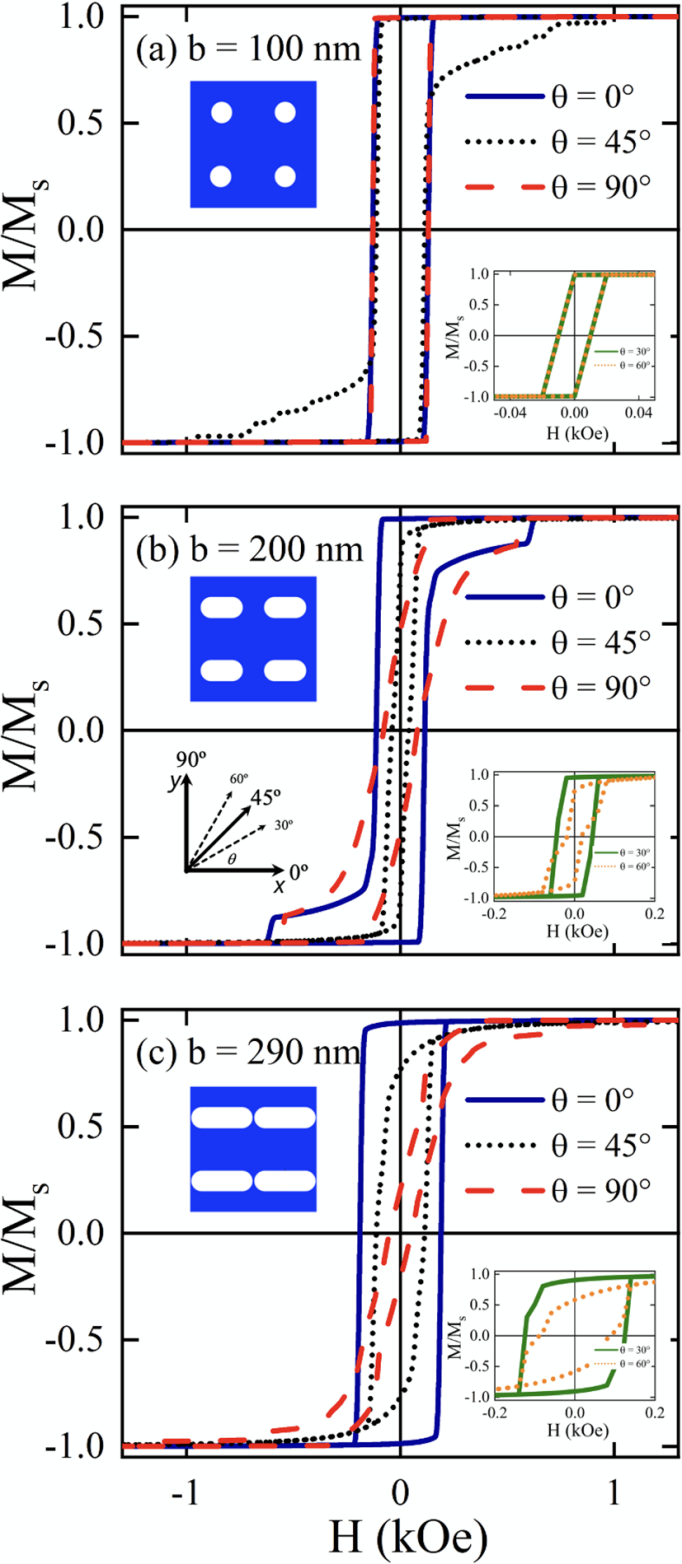
(Color online) Hysteresis loops for stadium-shaped antidot arrays when the magnetic field is applied along $\theta = 0^{\circ },45^{\circ },\text { and }90^{\circ }$ for (**a**) $b=100$ nm, (**b**) $b=200$ nm and (**c**) $b = 290$ nm. Insets correspond to the remaining cases $\theta = 30^{\circ } \text { and }60^{\circ }$. Snapshots of the stable magnetic configuration were taken at $H=-1.5$ kOe.

We start the study of static magnetic properties by investigating the hysteresis loops of antidot arrays varying both the geometry of the holes (*b*) and the angle ($\theta$) that forms the external magnetic field, *H*, with the x-axis (see Fig. 2). Figure 2a depicts the hysteresis loops for a circular antidot array ($b=a=100$ nm). In this figure we can see that cases $\theta =0^{\circ }$ and $\theta =90^{\circ }$ represented by blue solid and red dashed lines, respectively, are equivalent due to the symmetry along the *x*- and *y*-axes. There is also symmetry for cases $\theta =30^{\circ }$ and $\theta =60^{\circ }$ represented by green solid lines and orange dotted lines, respectively, manifested by the overlap of the hysteresis loops shown in the insert of Fig. 2a. Furthermore, the hysteresis curve representing $\theta =45^{\circ }$ (black dotted line) exhibits the same abrupt jump as that observed for $\theta =0^{\circ }$ and $\theta =90^{\circ }$, but in this case there are also several small jumps that indicate that the magnetization reversal occurs through more complex processes related to the nucleation, pinning and propagation of (super)domain walls^17^ due to the potential (inhomogeneous demagnetizing field) created by the antidots when the external magnetic field is applied along the diagonal.

**Figure 3:**
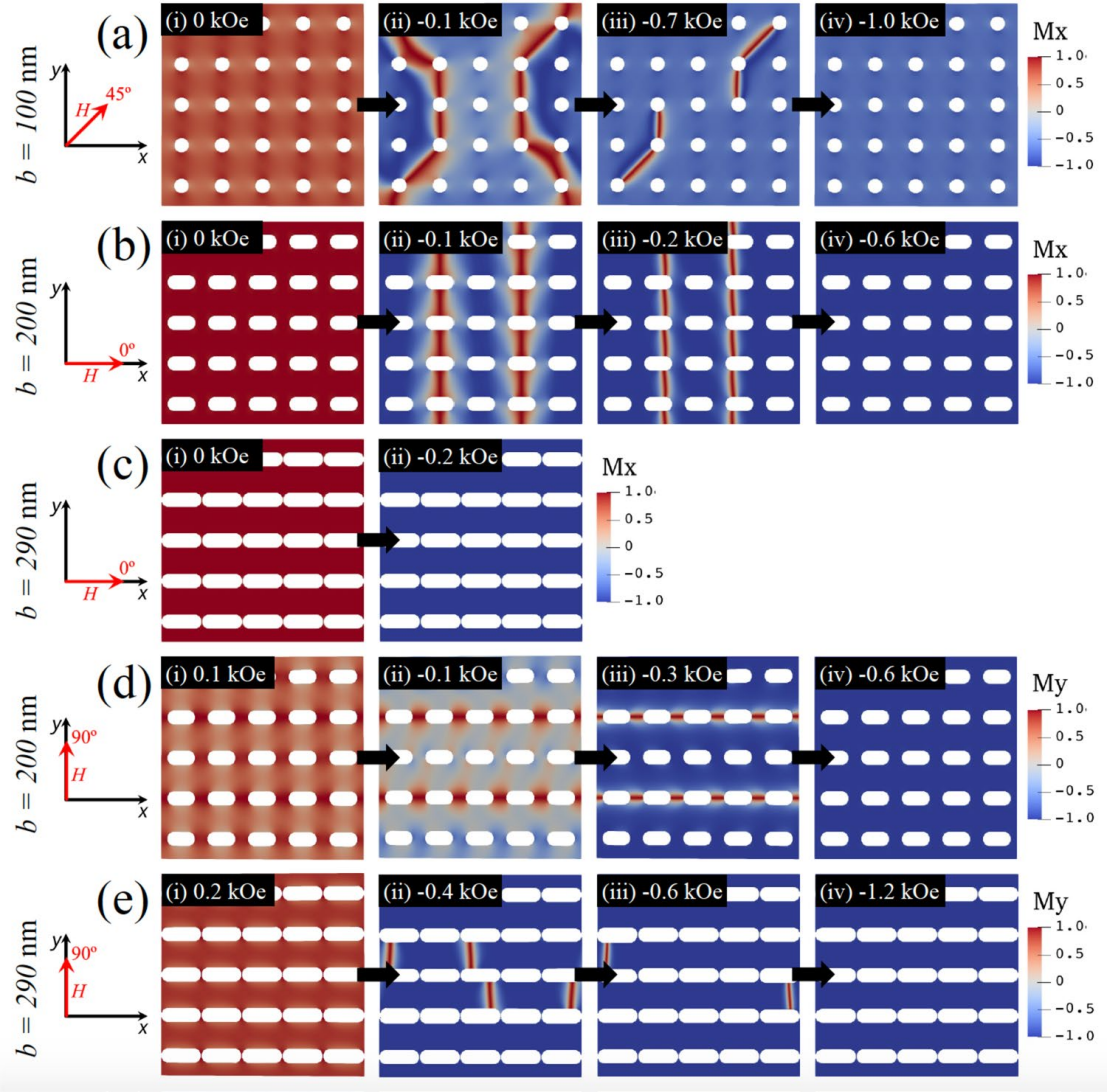
(Color online) Snapshots of the magnetization reversal process for (**a**) $b=100$ nm and $\theta =45^{\mathrm{o}}$, (**b**) $b=200$ nm and $\theta =0^{\mathrm{o}}$, (**c**) $b=290$ nm and $\theta =0^{\mathrm{o}}$, (**d**) $b=200$ nm and $\theta =90^{\mathrm{o}}$, and (**e**) $b=290$ nm and $\theta =90^{\mathrm{o}}$. The black arrows represent the temporal evolution of the reversal process and each snapshot corresponds to an instantaneous magnetic configuration at a given magnetic field shown in black boxes. The color bar corresponds to depicting of *x*-component of the magnetization for (**a**), (**b**) and (**c**), while for (**d**) and (**e**) corresponds to the *y*-component.

Figure 2b illustrate results for an antidot array with more elongated holes defined by $b = 200$ nm. In this case, the symmetry is broken, so that now the overlapping of the hysteresis loops for different angles that we observed in the previous case does not occur. In fact, we can see that there is an easy anisotropy axis along $\theta =0^{\circ }$ induced by the shape of holes. This behavior will continue while $b>a$. Therefore, we should not be surprised to lose the overlap of the hysteresis curves that we observed previously for $\theta =30^{\circ }$ and $\theta =60^{\circ }$. Note that for $\theta =45^{\circ }$ coercivity is the lowest of all, again manifesting a complex magnetization reversal process along this direction. Finally in Fig. 2c we show the antidot array with the longest hole allowed by our system, that is, $b=290$ nm. From this figure, the anisotropy induced along the elongation direction is evident, as well as that the system becomes like magnetic stripes. This can be seen from the hysteresis curves for $\theta =0^{\circ }$ and $\theta =30^{\circ }$, where the typical square-shaped hysteresis loops for elongated systems (stripes or wires) are observed. In the same way, when $\theta =90^{\circ }$ we obtain the typical hysteresis curve of a wire with a magnetic field applied perpendicular to its axis, that is, along its hard axis. Thus, this extreme case captures a physics similar to that exhibited by a nanowire^75–78^. To better understand the behavior observed in the hysteresis loops, in Fig. 3 we show snapshots of the magnetization reversal process of selected antidot arrays considering certain values of *b* and $\theta$. Figure 3a shows the snaphsots of the magnetization for $b=100$ nm and $\theta =45^{\mathrm{o}}$, considering different values of the magnetic field during the magnetization reversal process. As stated above, starting from the remanence state (Fig. 3a-i), the magnetization reversal occurs through a complex process that involves the nucleation and propagation of super domain walls, as shown in Fig. 3a-ii–a-iv. It should be noticed that this process is not homogeneous since segments of the super domain walls are pinned in the holes, as a consequence of the strong demagnetizing field that arises around the holes due to the accumulation of magnetic charges in this area. As the magnetic field increases in magnitude, the super domain wall is partially released until the reversal process is complete, as depicted in Fig. 3a-iv–a-vi.

Then, in Fig. 3b,c we examine arrays with more elongated holes, that is, $b=200$ nm and $b=290$ nm. Specifically, the magnetization reversal process for $b=200$ nm and $\theta =0^{\mathrm{o}}$ shows that, initially, there is an abrupt change in magnetization mediated by the propagation of several domain walls that are localized essentially in the vertical space existing between the holes. The domain walls are then annihilated, thus finishing the magnetization reversal process. It is important to note the difference with the case with $b=290$ nm, depicted in Fig. 3c, where the strong anisotropy induced by the shape of the holes produces a quick magnetization reversal process without evidencing the propagation of domain walls, at less, within the same time scale as in Fig. 3b. To understand the role that the induced anisotropy plays in the magnetization reversal process, in Fig. 3d,e we show that when the magnetic field is applied along the hard axis of the system ($\theta = 90^{\mathrm{o}}$), the competition between the exchange, dipolar and Zeeman energies give rise to a slower reversal process that considers the propagation of several domain walls, as shown in Figs. 3d-ii,d-iii and 3e-ii,e-iii. We also observe that for $b=200$ nm there is still enough magnetic material to nucleate domain walls along the *y* axis, which are eventually annihilated to finish the reversal process, unlike what happens when $b=290$ nm, where the shape of the holes is elongated enough to consider the system as separated nanostripes. In fact, in this case, the magnetization reversal process is carried out through the propagation of domain walls along the *x* axis, as shown in Fig. 3e-ii,e-iii, which is a characteristic behavior of nanowires.

**Figure 4:**
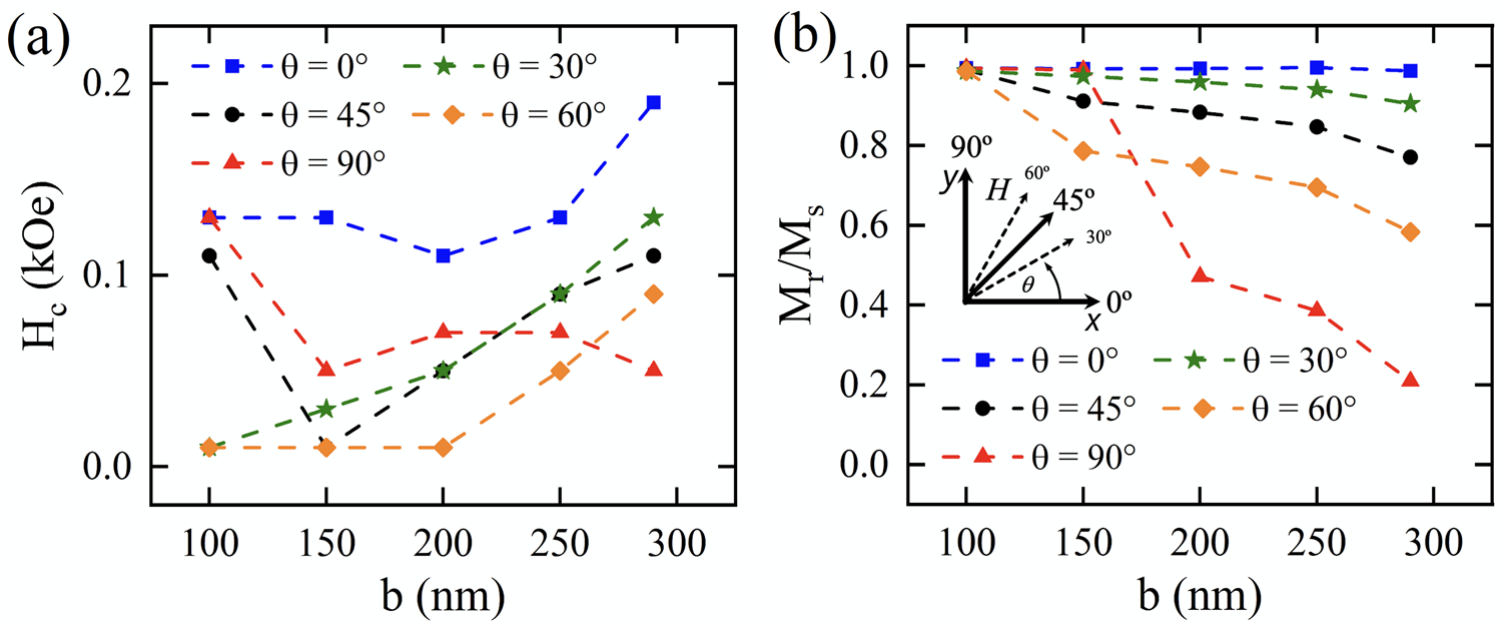
(Color online) (**a**) Coercivity and (**b**) normalized remanence as a function of the geometry of the holes (*b*) and the angle at which the external magnetic field is applied ($\theta$). The dashed lines are guides for eyes.

Our results for the static properties of the antidot arrays are summarized in Fig. 4, where the dependence of coercivity (Fig. 4a) and remanence (Fig. 4b) are shown as a function of the geometry of the holes (*b*) and the angle at which the external magnetic field is applied ($\theta$). From Fig. 4a we can see that coercivity is very susceptible to both parameters. This dependence becomes more evident as $\theta$ deviates from the extreme cases $\theta = 0^{\mathrm{o}}$ and $\theta = 90^{\mathrm{o}}$. We found a non-monotonically behavior that can be explained in terms of the complex variations that occur with the demagnetizing field when $b>a$, which promotes the propagation of super domain walls^17^ (see Figs. 2 and 3) and allows to control the pinning-depinning process of these, affecting to the coercivity of the system. On the other hand, the remanence is much less affected than the coercivity, showing a monotonic decrease as a function of b with a slope that increases with $\theta$. Notice that this behavior explains the decrease of the shape anisotropy energy as *b* increases. In fact, the remanence is maximal for $\theta =0 ^{\mathrm{o}}$ and minimal for $\theta =90 ^{\mathrm{o}}$, which implicitly defines an easy-axis anisotropy mediated by variations on *b* that quantifies the induced shape anisotropy as a function of hole elongations.

### Dynamic magnetic properties

**Figure 5:**
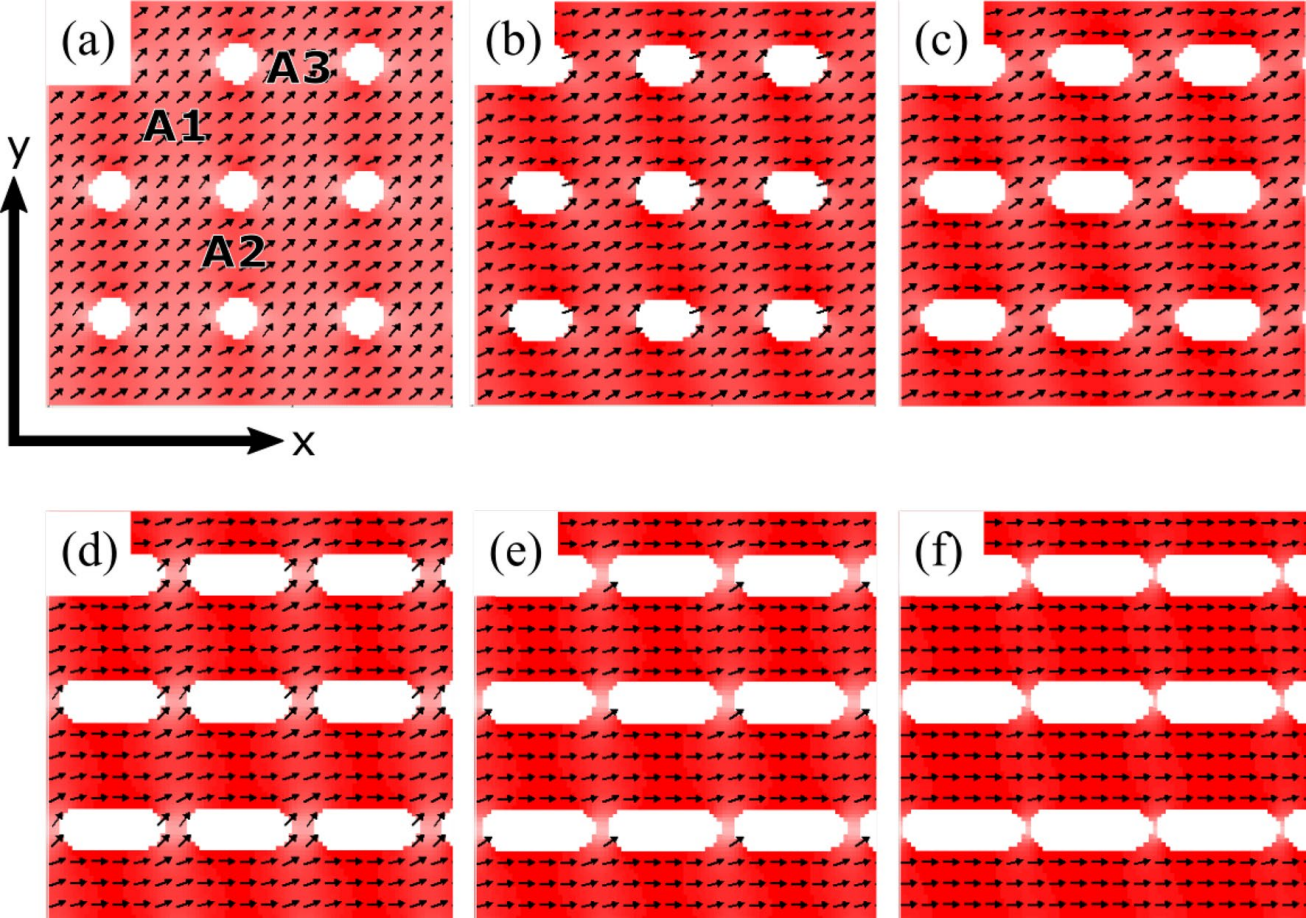
(Color online) Equilibrium magnetic configurations for (**a**) $b = 100$ nm, (**b**) $b=150$ nm, (**c**) $b=200$ nm, (**d**) $b = 250$ nm, (**e**) $b=270$ nm, and (**f**) $b=290$ nm. Pixels correspond to the spatial distribution of the in-plane component of magnetization, $M_x$. Red means the magnetic moments are oriented along the *x*-direction, while white indicates that the moments are oriented along the *y*-direction.

We start the study of dynamic properties by obtaining the equilibrium magnetic configurations of antidot arrays. This is done minimizing the energy of the system, in the absence of an external magnetic field (bias field), for the different values of *b*. Figure 5 shows the equilibrium configurations for antidot arrays ranging from circular holes ($b = 100$ nm) to stadium-shaped holes ($b > 100$ nm). Figure 5a shows the equilibrium configuration for an antidot array with circular holes, evidencing magnetic moments located near the edge of the holes that follow their curvature, which can be attributed to the shape anisotropy induced by the demagnetizing field of antidots. In this array we can distinguish three areas where the magnetic moments point along different directions: the central area, *region A1*, where the magnetic moments are oriented along the diagonal direction ($\theta \approx 45^{\mathrm{o}}$), the vertical region that separates the holes, *region A2*, where the magnetic moments are oriented at an angle slightly smaller than $45^{\mathrm{o}}$, and the horizontal region that separates the holes, *region A3*, where the magnetic moments are oriented at a slightly higher angle than $45^{\mathrm{o}}$. Similar equilibrium configurations are also found in Fig. 5b–f ($b>a$), but in such arrays it must be keep in mind that the A2 regions are larger than the A3 regions, because the *b* parameter ranges from 100 to 290 nm.

**Figure 6:**
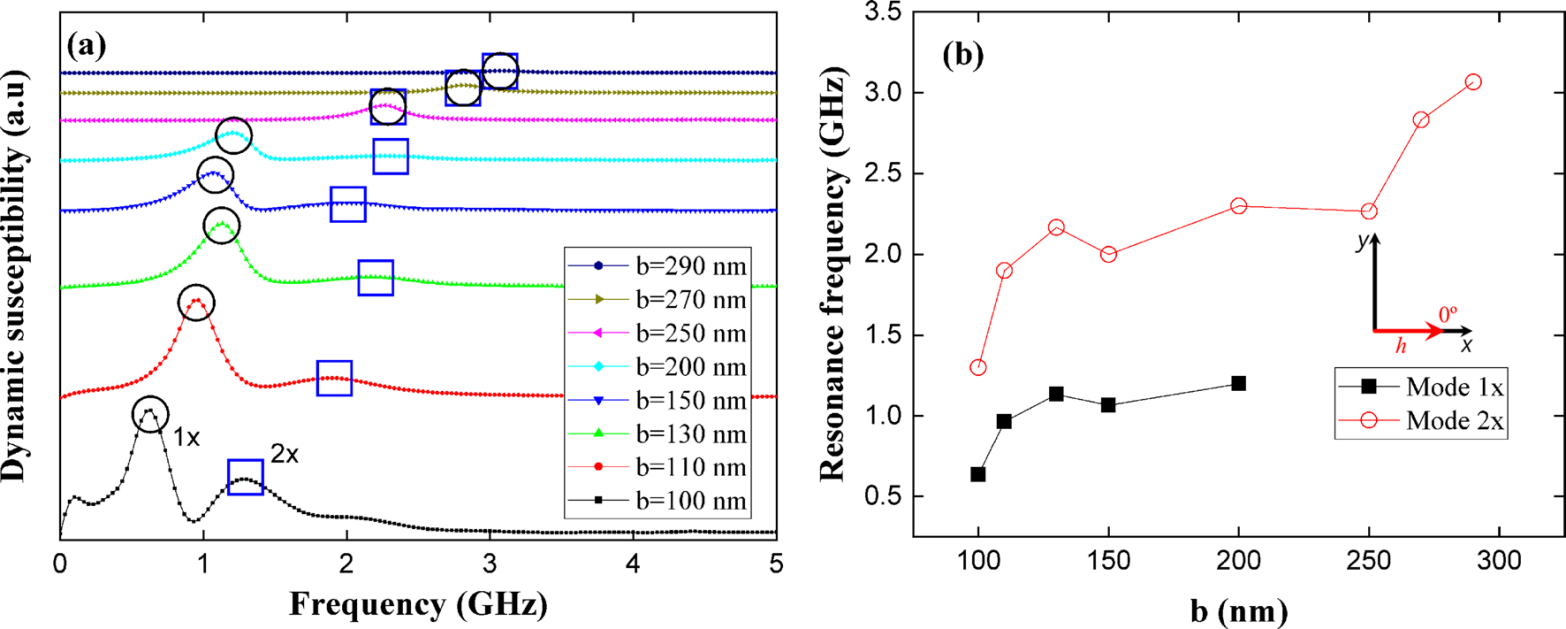
(Color online) (**a**) Dynamic susceptibility of stadium-shaped antidot arrays with different values of *b* parameter, when a magnetic pulse is applied along the *x*-axis. (**b**) Evolution of resonance frequencies of stadium-shaped antidot arrays as a function of *b* parameter.

We continue analyzing the dynamic magnetic response of the sample when a small exciting magnetic pulse is applied to its equilibrium configuration (for each value of *b* ) along the *x*- and *y*-direction. In Fig. 6a we depict the imaginary part of the magnetic susceptibility and resonance modes when a magnetic pulse is applied along the *x*-axis. This figure evidence two resonance peaks (1*x* and 2*x* peaks) for *b* varying between 100 and 200 nm. However, when we further increase *b* ($b > 200$ nm), we can see only one resonance peak due to mode 1*x* disappears (see Fig. 6b). This phenomenon, which occurs for $b > 200$ nm, is mainly due to the precession of the magnetic moments located within area A3, as we will show below.

**Figure 7:**
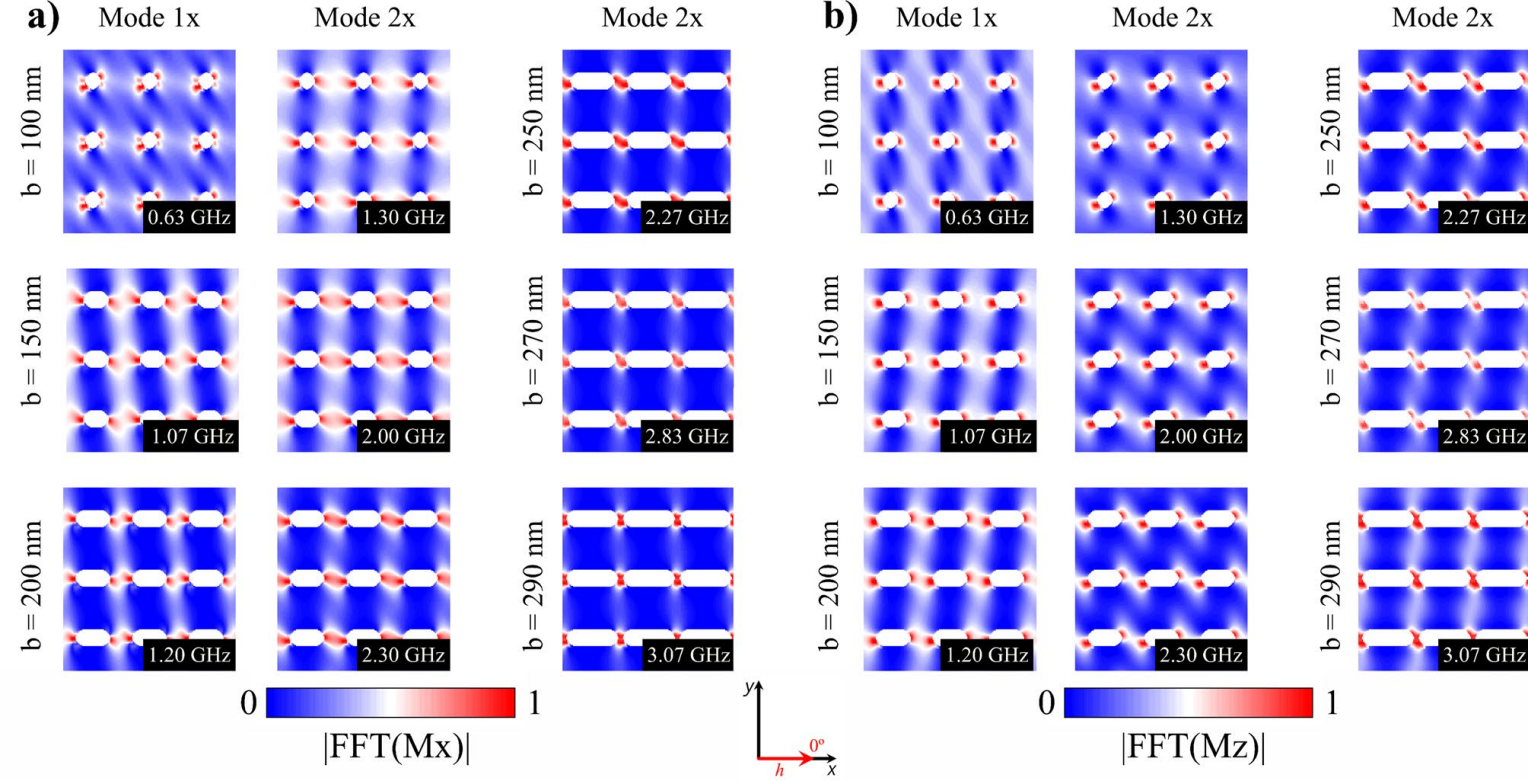
(Color online) **a** *x*-component and **b** *z*-component of the spatial distribution of the dynamic susceptibility for each resonance frequency of stadium-shaped antidot arrays when a magnetic pulse was applied along the *x*-axis. The bright part (red) reflects high spin precession amplitude and the dark part (blue) corresponds to zero amplitude.

It is interesting to explore the spatial distribution of magnetic moments for the resonance modes, in order to exactly know which spatial regions are coherently excited. Thus, Fig. 7 shows these spatial distributions for both the *x*- (Fig. 7a) and *z*-component (Fig. 7b) of the magnetization field for different *b* values. From this figure we can see how the resonance modes are associated with the response of the equilibrium configurations to the external magnetic pulse 1*x* (low frequency) and 2*x* (high frequency). The resonance modes are originated mainly on the perturbation of the magnetic moments located in the areas A1 and A3 of the sample. Moreover, the prevalent mode 2*x* (remember that peak 1*x* disappears for $b>200$ nm) originates mainly on the perturbation of the magnetic moments located in A3, as can be seen in Fig. 7a,b. This behavior is essentially a consequence of the strong shape anisotropy mediated by the elongated holes, which favors the spins alignment along the *x*-direction, and can be explained as follows: By increasing *b*, the spatial distribution of the resonant modes 1*x* and 2*x* gradually became similar because the population of spins belonging to the area A2 increases as well. When elongating the holes, such magnetic moments prefer to point along the *x*-direction, the same direction of the magnetic pulse, finally suppressing spin excitations in A2 (recall that linear spin wave theory establishes that coherent magnetic excitations are created in a plane perpendicular to the magnetization). Thus, for the pulse applied along the *x*-direction, larger *b* values promote the excitation of magnetic moments belonging only to A3 and the energy needed to excite them is higher too, which explains the suppression of the low energy mode (mode 1*x*) in Fig. 6b.

**Figure 8:**
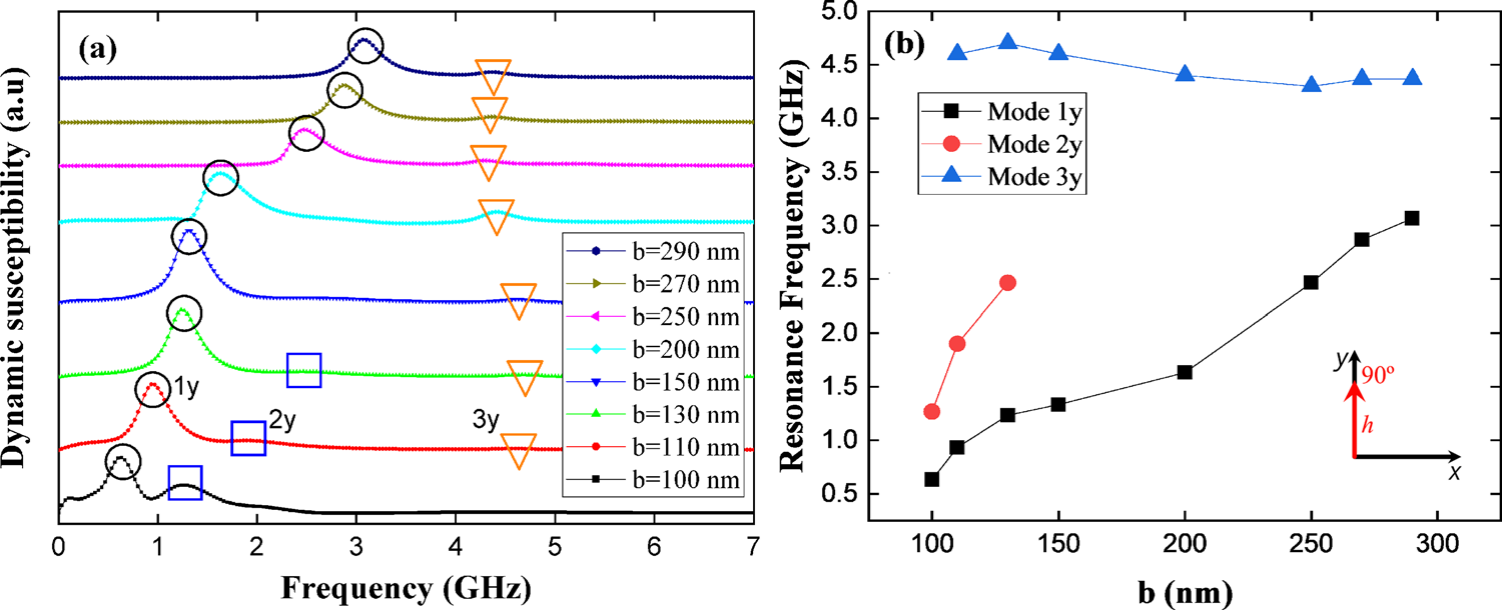
(Color online) (**a**) Dynamic susceptibility of stadium-shaped antidot arrays with different values of *b* parameter, when a magnetic pulse is applied along the *y*-axis. (**b**) Evolution of resonance frequencies of stadium-shaped antidot arrays as a function of *b* parameter.

**Figure 9:**
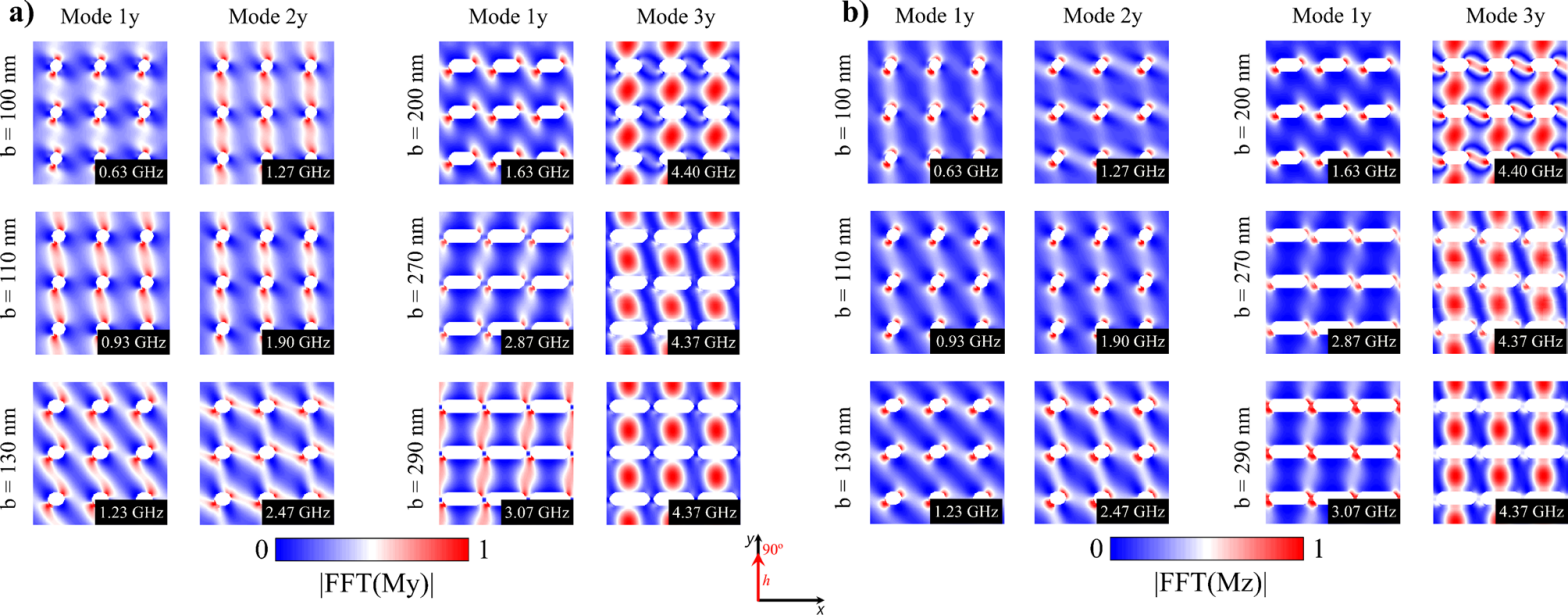
(Color online) **a** *x*-component and **b** *z*-component of the spatial distribution of the dynamic susceptibility for each resonance frequency of stadium-shaped antidot arrays when a magnetic pulse was applied along the y-axis. The bright part (red) reflects high spin precession amplitude and the dark part (blue) corresponds to zero amplitude.

Following the dynamic study, we now explore the magnetic response of the system when the magnetic pulse is applied in the *y*-direction. Fig. 8 shows the dynamic susceptibility in this case. From this figure we can observe three resonance peaks (1*y*, 2*y*, and 3*y*) according to the range of *b*. Specifically, mode 1*y* holds for the entire range of *b*, while mode 2*y* holds for $100<b<130$ nm and disappears for $b>130$ nm, and mode 3*y* (the highest frequency mode) appears when introducing the asymmetry in the system, i.e., for $b>100$ nm. Furthermore, Fig. 8b shows the evolution of these resonance frequencies as a function of *b*, evidencing that the frequency associated to peak 1*y* monotonically increases with increasing *b*, while the frequency corresponding to peak 2*y* has the same behavior until the mode disappears. This occurs because the population of spins belonging to A3 (where mode 2*y* localizes for $b = 130$ nm) decreases as *b* increases, as can be seen from Fig. 9a,b, where the *y*- and out-of-plane-components of the magnetization field of modes 1*y*, 2*y*, and 3*y* are depicted. On the other hand, mode 3*y* is a higher frequency mode whose frequency slightly decreases for larger *b* values. Since the pulse is applied along the *y*-direction, the high energy spectrum means that for the entire range of *b* there is a still relevant *y*-component of the magnetization field, given by the magnetic ground state (see Fig. 5). Once *b* becomes bigger, the induced shape anisotropy strongly aligns the spins along the *x*-direction and the energy needed to excite 3*y* mode slightly decreases and then is more or less constant. Therefore, when the pulse is applied along a hard magnetization axis, the resonance frequency is determined mainly by the spin population in A2.

In summary, the five excited modes studied depend directly on *b*, as can be seen from Figs. 6, 8, and 9. In fact, the source of modes 1*x* and 2*x* can be explained as follows: modes 1*x* and 2*x* have a common origin, which is the excitation of magnetic moments belonging at areas A1 and A3, as shown in Fig. 7. As long as *b* increases, the area A2 grows, and then the spin wave modes localize at A3 area due to the induced shape anisotropy tilts the spins towards the *x*-axis. This implies that higher frequencies are needed to excite a given spin wave mode, and consequently, at larger *b* values, the resonance frequency increases as well. On the other hand, as shown in Fig. 9a,b, 1*y*, 2*y*, and 3*y* modes have a different origin. Indeed, modes 1*y* and 2*y* originated on excited magnetic moments belonging mainly to A1 and A2, for $b=130$ nm, 2*y* mode localizes at A3. This region becomes smaller as *b* increases, finally suppressing this mode. On the other hand, 3*y* mode originated on excited magnetic moments belonging to A2, which indeed becomes larger as *b* approaches 290 nm. Therefore, for small *b* values ($b<130$) there is still a considerable population of spins pointing along the *y*-direction, which opposes to the spin wave excitation; while for $b > 130$ nm, A2 is bigger and most of the spins in such area point along the *x*-direction. Since the pulse is applied along the *y*-direction, the energy needed to excite the spin wave modes slightly decreases compared with the above cases ($b < 130$ nm) and then holds for the rest of b studied values. It is important to notice that Figs. 6, 8, and 9 agree with linear spin wave theory^61^ in the sense that, due to the normalization of the magnetization vector, the magnetic excitations live in a plane perpendicular to the direction of the magnetization and then must be excited with a perpendicular magnetic field. Therefore, the *x*-component of the magnetic field excites the *z*- or *y*- components of the magnetization, and the *y*-component of the magnetic field excites the *x*- or *z*-components of the magnetization. That is why different spin wave modes are excited when applying the pulse along the *x*- or *y*-direction, even more considering that the *b* parameter controls the population of magnetic moments pointing along the *x*-direction.

## Conclusions

In conclusion, we studied both the static and dynamic properties of magnetic antidot arrays with holes whose geometry ranged from a circle to a stadium shape.

Concerning the static properties we found that coercivity is highly sensitive to the geometry of the holes. In particular, when $b>a$, changes in coercivity can be explained in terms of the strong shape anisotropy induced by elongated holes. The critical case occurs when $b=290$ nm, where the system behaves like an array of non-interacting magnetic stripes. This fact is confirmed by analyzing the magnetization reversal modes, since they mimic very nicely those observed in nanowires. We also found that remanence is generally less affected by the hole geometry. This is a direct consequence of the low mobility of the nucleated domain walls due to the pinning potential created by holes that slow down the magnetization reversal process, inhibiting abrupt changes in the remanence when we vary *b*. Once the shape anisotropy is strong enough, the pinning potential weakens, and then the remanence decreases.

In the dynamic section we found the resonance modes as a function of the geometry of the holes and the angle at which the external magnetic pulse was applied. In fact, when the perturbation was applied along the hole axis, there is a critical *b* value for which the low energy mode vanishes, keeping only the higher one in the whole range of *b*. Thus, it is possible to control not only the magnitude of the resonance peaks, but also the number of peaks. In the same way, when the magnetic pulse is applied perpendicular to the hole axis, one of the three resonance modes disappears. Notice that, although our study was performed in an ideal system with no edge roughness, the inclusion of such defects should modify both the shape and the number of resonance modes^25,31,56,58,79^. Although, we expect that due to the significant increase of the unidirectional anisotropy, the changes due to the extrinsic defects are minor or despicable. Finally, the suppressing of one resonance peak when the pulse is applied along the *y*- or *x*-direction, can be associated to an intrinsic feature of potential magnonic filters. Thus, our results could open up new opportunities to improve spintronics, electronics, and even magnonics devices.

## Supplementary information

Supplementary Information 1.
