# Supplementary material for: Dynamic and static properties of stadium-shaped antidot arrays
Source: Sci Rep. 2020 Nov 18;10:20024. doi: 10.1038/s41598-020-77074-2 (PMC7674446; doi:10.1038/s41598-020-77074-2)
Supplement: Supplementary file 1 — Supplementary Information 1. [file 41598_2020_77074_MOESM1_ESM.pdf]

SUPPLEMENTARY INFORMATION

Dynamic and static properties of stadium-shaped antidot arrays

E. Saavedra[1], R. M. Corona[1,4], N. Vidal-Silva[2,4,*], J. L. Palma[3,4], D. Altbir[1,4], and J. Escrig[1,4]

[1]*Departamento de Física, Universidad de Santiago de Chile, Avda. Ecuador 3493, 9170124 Santiago, Chile*

[2]*Departamento de Ciencias Físicas, Universidad de La Frontera, Casilla 54-D, 4811186 Temuco, Chile*

[3]*Universidad Central de Chile, Avda. Santa Isabel 1186, 8330601 Santiago, Chile*

[4]*Center for the Development of Nanoscience and Nanotechnology, Avda. Libertador Bernardo O'Higgins 3363, 9170124 Santiago, Chile and*

[*]*nicolas.vidal@ufrontera.cl*

(Dated: September 29, 2020)

I. DEPENDENCE OF THE DYNAMIC SUSCEPTIBILITY ON THE α PARAMETER

Here we show that our results have been obtained with a proper damping α value, as stated in the main text. In Fig. S1 we show the dynamic susceptibility as a function of the frequency.

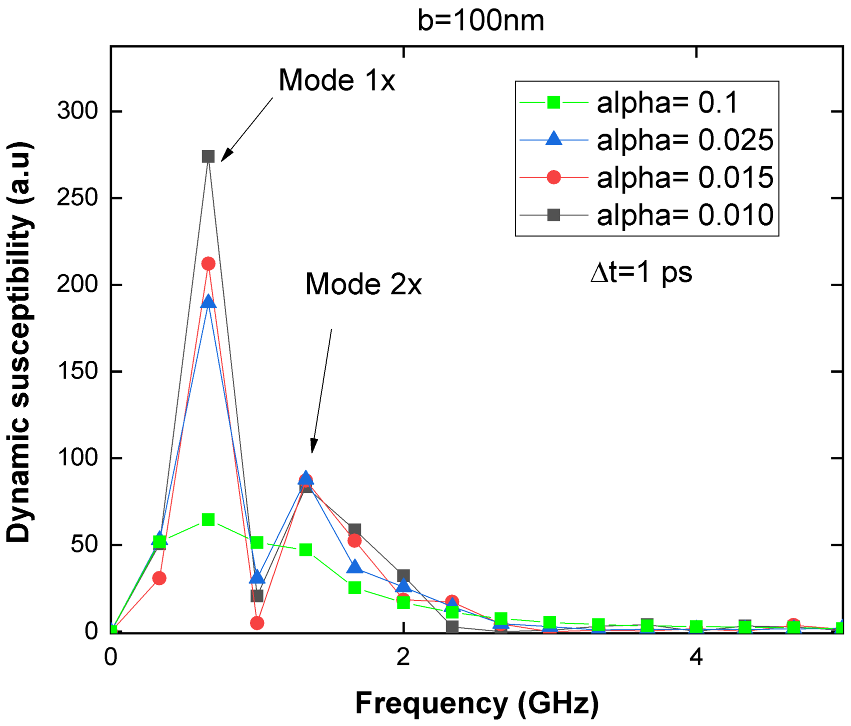

FIG. S1. (Color online) Dynamic susceptibility of stadium-shaped antidot arrays with b=100 nm, when a magnetic pulse is applied along the x-axis and for different α values.

From this figure can be directly seen that the α value is a crucial parameter when studying dynamic properties since by no choosing a proper value, relevant information can be missed. This is the case for $\alpha = 0.1$, where it is evident the absence of modes $1x$ and $2x$ because the system is overdamped. However, when decreasing α, the system does show both resonance modes and, more importantly, the position of the peaks is unaltered. This behavior holds for all $\alpha < 0.1$ in the range of tested values, which gives us the possibility to choose the proper α value. Therefore, since we are primarily interested in the position of the peaks, then we set $\alpha = 0.025$, as stated in the main text.
